# Funding for Refugee Health Research From the National Institutes of Health Between 2000 and 2020

**DOI:** 10.1001/jamanetworkopen.2023.50837

**Published:** 2024-01-10

**Authors:** Mehak Kaur, Lana Bridi, Dahlia Kaki, Behnan Albahsahli, Nissma Bencheikh, Altaf Saadi, Gretchen Bandoli, Cheryl A.M. Anderson, Alissa Bernstein Sideman, Tala Al-Rousan

**Affiliations:** 1 Department of Population and Public Health Sciences, Keck School of Medicine, University of Southern California, Los Angeles; 2 School of Medicine, University of California, San Diego, La Jolla; 3 School of Medicine, University of California, San Francisco; 4 Herbert Wertheim School of Public Health and Human Longevity Science, University of California, San Diego, La Jolla; 5 Department of Neurology, Harvard Medical School, Boston, Massachusetts; 6 Philip R. Lee Institute for Health Policy Studies, University of California, San Francisco

## Abstract

**Importance:**

The US has historically resettled more refugees than any other country, with over 3.5 million refugees since 1980. The National Institutes of Health (NIH) is the largest public funder of biomedical research and development, but its role in mitigating many health disparities refugees experience through its funded research remains unknown.

**Objective:**

To examine the NIH’s research funding patterns on refugee health research over the last 2 decades.

**Design, Setting, and Participants:**

Secondary analysis of NIH-funded grants between 2000 and 2020 using a cross-sectional study design. The NIH Research Portfolio Online Reporting Tools database was used to find relevant grants. Data were analyzed from November 2021 to September 2022.

**Main Outcomes and Measures:**

NIH grants awarded by year, state, grant type, research area, funding institute, grant duration, and amount funded.

**Results:**

Of 1.7 million NIH grants funded over the 20-year study period, only 78 addressed refugee health. Funded grants were mostly training grants (23 grants [29%]), followed by hypothesis-driven research (R01 grants; 22 grants [28%]), pilot or preliminary investigation proposals (13 grants [17%]), and other types of grants (20 grants [26%]). The most studied research domain was mental health (36 grants [46%]), followed by refugee family dynamics and women’s and children’s health (14 grants [18%]). A total of 26 grants (33%) were funded by the National Institute of Mental Health and 15 (19%) were funded by the National Institute of Child Health and Human Development. Most grants were US-based (60 grants [76%]) and the state of Massachusetts received the greatest amount of funding ($14 825 852 [18%]). In 2020, the NIH allocated about $2.3 million to refugee health research, or less than 0.01% of its $42 billion budget that year. The number of grants funded in each time period did not always reflect changes in the number of refugees resettled in the US over the years.

**Conclusions and Relevance:**

This cross-sectional study found that there remain significant gaps in the understanding of and interventions in the health research needs of refugees locally and along the migratory route. To close these gaps, the NIH should increase its investments in comprehensive studies assessing the physical, mental, and social well-being of this expanding population. This can be achieved by ensuring that all NIH institutes allocate budgets specifically for refugee health research and extend support for the training of refugee researchers.

## Key Points

**Question:**

What is the status of refugee health research funding within and across all the National Institutes of Health (NIH) over 20 years?

**Findings:**

This cross-sectional study found a total of 78 grants that focused on researching refugee health; almost half of this research focused on mental health (46%), with the National Institute of Mental Health funding most grants (33%). The total amount spent was $81.2 million (2000 to 2020); for context, the NIH spent less than 0.01% of its 2020 budget on refugee health research.

**Meaning:**

Despite a plethora of documented health disparities among refugees and an exponentially growing population of refugees, these findings suggest the NIH provided minimal funding to support refugee health research.

## Teaser

This cross-sectional study evaluates the National Institutes of Health’s funding patterns in refugee health research over the last 2 decades.

## Introduction

One in 74 people in the world are forcibly displaced, which includes 40.7 million refugees and asylum seekers, roughly equivalent to the population of Canada.^1^ Over the past decade alone, the number of refugees worldwide has more than doubled, resulting in 108.4 million forcibly displaced people, the highest number on record.^1,2^ This number is expected to grow due to ongoing geopolitical turmoil, climate-induced migration, and rising economic inequality. The United Nations High Commissioner for Refugees (UNHCR) defines a refugee as a person who was forced to leave their own country because of war, persecution, or widespread violence.^3^ All refugees were asylum seekers at one point, having sought protection from persecution while waiting to receive a decision on their asylum claim. These distinctions are legal ones, but both encompass the similar experience of seeking refuge in a new country because they are at risk of persecution in their home country.

Refugees have the human right to health, and countries have obligations to provide refugees and forcibly displaced people health care services. This is delineated in the 1951 Convention Relating to the Status of Refugees and its 1967 Protocol,^4^ and is important as prior research has shown a higher prevalence of various neuropsychiatric disorders such as posttraumatic stress disorder (PTSD), depression, and head trauma as well as chronic diseases such as cardiovascular disease.^5,6,7^ Yet despite both these protections and known health needs, they experience significant health disparities.^8^ They face health care barriers including lack of access and interrupted health care coverage, difficulty navigating complex systems, lack of culturally and linguistically concordant care, discrimination, and poor health care utilization.^9,10,11,12,13^ These health care barriers are compounded by a heightened exposure to premigratory, midmigratory, and postmigratory mental and physical trauma, loss of social networks, barriers to accessing care along the migratory route, and other stressors that contribute to cycles of health disparities.^14^ There is growing literature demonstrating the transgenerational transmission of health disparities from refugees to their children.^15,16,17^ Yet many questions remain about how best to care for this highly trauma-exposed population given a dearth of evidence-based clinical guidelines and interventions.^18^

Conducting refugee health research is particularly challenging because few public health systems collect data on migration history and experiences. Refugee populations are often studied within their racial and ethnic groups without inquiry into their displacement history or considering race as a social construct.^19,20,21,22^ Designing novel health interventions to address health disparities in the minoritized refugee population requires a deep understanding of various contextual factors such as wars or other trauma exposure in the country of origin, experiences during migration, resettlement community politics and capacities, education level, and social support systems, among others. Refugee health research is needed to assess these interplaying factors and formulate evidence-based recommendations allowing for achieving optimal health over the life course.^23,24,25,26^

The National Institutes of Health (NIH), which invests almost $48 billion in health research every year, is the largest public funder of health research in the world and provides a lifeline pathway for rectifying this knowledge gap.^27^ Within the lens of a human rights framework, the NIH’s research mission should elevate not only research that considers the public interest within a broader, international context but also with a focus on those marginalized and at risk of human rights violations domestically and globally. This is aligned with their larger mission to support research on health disparities and health inequities.^28^ Yet, to date, there has been no formal evaluation of NIH funding allocated toward refugee health research in the US, which has welcomed almost 3.5 million refugees since the Refugee Act of 1980, and in 2021 received the largest number of individual refugee applications worldwide.^29,30^ This study seeks to address this gap in the literature and assess NIH research funding for refugee health from 2000 to 2020. This study was designed to (1) conduct a detailed analysis of NIH funding in refugee health research over the last 2 decades and (2) identify important future research directions, including the role of the NIH in establishing and funding a refugee health research agenda.

## Methods

### Data Collection

A cross-sectional study was performed in October 2021, following the Strengthening the Reporting of Observational Studies in Epidemiology (STROBE) reporting guideline.^31^ The Institutional Review Board at the University of California San Diego determined that no ethical approval was required. Informed consent was not required because there were no human participants in this study. We defined refugee health research as any study that explicitly addresses the health or health care of people who identify as asylum seekers, refugees, and refugees resettled in the US. We used the NIH Research Portfolio Online Reporting Tools (RePORT), a publicly available database, to analyze all refugee health research grants between 2000 and 2020. We used the search terms *refugee*, *asylum seeker*, *forced migration*, *migrant*, *emigrant*, *refugee*, and *United States* separately to find all the relevant grants. The first author (M.K.) conducted all the searches separately and only combined the terms *refugee* and *United States* to look for grants specifically funded in the US. These terms were chosen based on commonly used terms for refugees according to the United Nations definition of displaced communities and consensus of all coauthors who are experts in the field (T.A., A.S., C.A.M.).

Grants were excluded if they mentioned the keywords but did not study refugee health (eg, grants that mentioned refugee populations as examples but were not the intended study group). Similarly, projects that returned nonrelated topics were also excluded (eg, the keyword *migrant* yielded grants that were related to migratory cells in the human body) (eTable 1 in Supplement 1). Additionally, grants that only had their title but were missing abstracts and/or funding information on the database were also excluded.

We recorded the grant number, grant type, NIH institute or center, grant title, dollar amount awarded, state of the institute that received funding, and grant abstract. Using the abstract, we also recorded the grant’s primary research area designation, type of data collection, involvement of community engagement practices, and whether it was spent locally or internationally. Community engagement was defined as an approach specifically designed to interact with groups of people that are related by affiliation, geography, or a shared interest to address issues affecting their well-being.^32,33^ Therefore, community engagement was determined based on whether the researchers collaborated directly with the refugee group they were studying or had partners from that community that assisted with the research process. Two authors independently determined the relevance of each grant and abstracted the data, and any disagreements were resolved by a third author (M.K., B.A., N.B., and T.A.). Data on the number of refugees admitted yearly were combined using the yearly admissions data by the Migration Policy Institute which were then trended over the 20-year period to compare with the number of grants.^34^ All data were collated into a database in Excel, version 16.79.2 (Microsoft) for data management and analysis.

### Data Analysis

Grants were categorized according to the following 6 primary research areas: (1) mental health; (2) refugee family dynamics and women’s and children’s health; (3) HIV and/or substance use; (4) health care delivery, including intervention and implementation science; (5) health disparities with other immigrants or the host population as comparison groups; and (6) chronic disease. These categories were determined based on the common research areas that emerged from the abstracts of all the selected grants. Studies that spanned multiple research areas were categorized according to their primary research area based on their main study aim. We summarized the total number of grants funded by each NIH institute or center, the number of each type of grant, the amount of money allotted to each grant, the number of grants by primary research area, type of data collection (primary vs secondary), involvement of community engagement practices, and the location (state) of the institute that received funding along with the location of the research project (local or international). Data were analyzed from November 2021 to September 2022.

## Results

### Refugee Health Research Grants Funded by the NIH, 2000 to 2020

**Figure 1.**
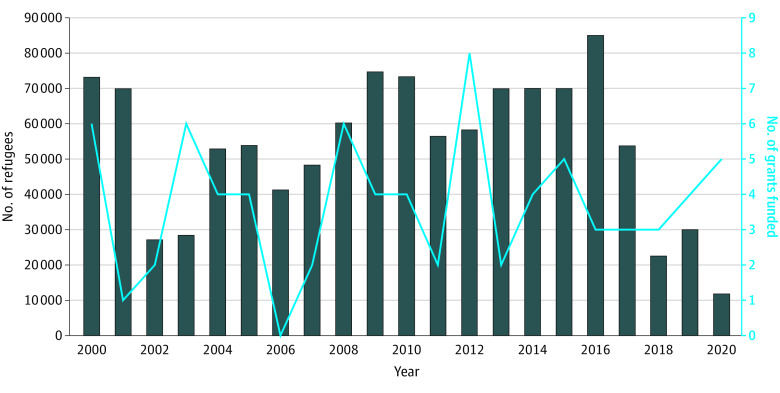
Number of Refugees Admitted and Grants Funded by Year of Admission Dark blue bars indicate number of refugees admitted; light blue line indicates number of grants.

Our keyword search on the NIH RePORT database returned 768 unique refugee health research grants out of more than 1.7 million NIH projects funded between 2000 and 2020. Of those, only 78 grants met the inclusion criteria (eFigure 1 in Supplement 1). The rest were excluded for various reasons including grants citing refugee health research conducted previously; using the keyword *refugee* but not actually studying refugees in the research aims; grants funding centers, cores, and conferences for refugee health but not directly doing refugee health research; and more (see eTable 1 in Supplement 1). Notably, multiple grants used the identifiers *refugees* or *migrants* interchangeably, despite legal differences between these 2 populations. Our study also identified multiple grants misusing the identifier of *refugee health research* when not conducting research with or for refugee populations based on the information the researchers provided in their abstract. To date, the number of refugee-related grants varied by funding period, with the largest number (8 grants) funded in 2012, and the smallest number (0 grants) funded in 2006 (Figure 1). The cumulative number of refugees admitted in the US since 2000 was 1 130 479. The number of grants funded each year did not always reflect changes in the number of refugees resettled in the US over the years (Figure 1).

### Refugee Health Research by Primary Research Area

Of the 78 funded grants, the primary research focus was mental health in 36 grants (46%), refugee family dynamics and women’s and children’s health (including intergenerational studies) in 14 grants (18%), HIV and/or substance use in 8 grants (10%), health care delivery (including interventions) in 8 grants (10%), and 12 grants (15%) in other topics. Other research areas included health disparities (8 grants [10%]) and chronic disease (4 grants [5%]) (Table 1). Of the 78 funded grants, the majority conducted primary data collection (70 grants [90%]) and integrated community engagement practices engaged in their research (69 grants [89%]).

**Table 1.** National Institutes of Health Funding for Refugee Health Research by Primary Research Area

Primary research area	Grants, No. (%)						Funding awarded 2000 to 2020, $ (%)
	Awarded 2000 to 2020	Investigator-initiated research		Training grants^b^		Other research^e^	
		Pilot^a^	R01	Junior^c^	Other^d^		
Mental health	36 (46)	6 (46)	13 (59)	13 (59)	0	4 (20)	34 184 089 (42)
Refugee family dynamics and women’s and children’s health	14 (18)	2 (15)	3 (14)	6 (27)	0	2 (10)	10 003 400 (12)
Health disparities	8 (10)	0	0	0	0	8 (40)	16 219 063 (20)
HIV and/or substance use	8 (10)	3 (23)	3 (14)	1 (5)	1 (100)	1 (5)	6 975 693 (9)
Healthcare delivery	8 (10)	2 (15)	2 (9)	1 (5)	0	3 (15)	5 190 419 (6)
Chronic diseases	4 (5)	0	1 (5)	1 (5)	0	2 (10)	8 654 066 (11)
Total	78 (100)	13 (17)	22 (28)	22 (28)	1 (1)	20 (26)	81 226 730 (100)

^a^ Grant types R03, R21, and R34.

^b^ Career Development Award.

^c^ Grant types F31, F32, K01, K23, and T32.

^d^ Grant type K24.

^e^ Grant types P20, R15, R24, R43, T37, U01, U13, U48, U54, and U58.

### Refugee Health Research by Institute or Center

The National Institute of Mental Health (NIMH) and the National Institute of Child Health and Human Development (NICHD) together funded over 50% of all selected grants (Table 2). The NIMH funded 26 grants (33%) and the NICHD funded 15 grants (19%). Sixteen other institutes and centers funded at least 1 refugee health research grant. Of the 78 grants, 13 (17%) were pilot grants (small exploratory grants), 22 (28%) were R01s (grants funding hypothesis-driven health research),^28^ 23 (29%) were junior training grants (small grants funding early career investigators), and the remaining 20 (26%) were other types of research grants, which included P20 grants that fund interdisciplinary programs or centers (Table 2).^35,36^ The average duration of a grant varied based on the type of grant. Research grants such as the T37 (18 years) and U54 (15 years) had the longest duration of funding as opposed to junior training grants such as the T32, which had an average duration of 6 years. Investigator-initiated research grants had the shortest duration (R01: 4 years; R03, R21, and R34: 2 years) (eFigure 2 in Supplement 1).

**Table 2.** National Institutes of Health Funding for Refugee Health Research by Institute or Center

Institute	Grants, No. (%)						Funding awarded 2000 to 2020, $ (%)
	Awarded 2000 to 2020	Investigator initiated research		Training grants^b^		Other research^e^	
		Pilot^a^	R01	Junior^c^	Other^d^		
National Institute of Mental Health	26 (33)	4 (31)	9 (41)	13 (59)	0	0	25 840 396 (32)
Eunice Kennedy Shriver National Institute of Child Health and Human Development	15 (19)	3 (23)	4 (18)	5 (23)	1 (100)	2 (10)	12 855 294 (16)
National Institute on Minority Health and Health Disparities	10 (13)	0	2 (9)	0	0	8 (40)	18 397 349 (23)
National Institute of Nursing Research	5 (6)	1 (8)	1 (5)	1 (5)	0	2 (10)	3 128 881 (4)
National Cancer Institute	3 (4)	1 (8)	1 (5)	0	0	1 (5)	8 092 002 (10)
National Heart, Lung, and Blood Institute	3 (4)	0	1 (5)	1 (5)	0	1 (5)	2 358 230 (3)
National Institute on Drug Abuse	3 (4)	2 (15)	1 (5)	0	0	0	1 209 095 (2)
Fogarty International Center	3 (4)	1 (8)	0	1 (5)	0	1 (5)	1 018 923 (1)
National Center for Chronic Disease Prevention and Health Promotion	2 (3)	0	0	0	0	2 (10)	3 951 701 (5)
Center for Global Health	2 (3)	0	0	0	0	2 (10)	813 220 (1)
National Institute of Dental and Craniofacial Research	1 (1)	0	1 (5)	0	0	0	1 530 130 (2)
National Library of Medicine	1 (1)	0	0	0	0	1 (5)	483 528 (1)
National Institute on Alcohol Abuse and Alcoholism	1 (1)	0	1 (5)	0	0	0	443 881 (1)
National Institute for Occupational Safety and Health	1 (1)	0	1 (5)	0	0	0	417 900 (1)
National Institute of Neurological Disorders and Stroke	1 (1)	0	0	1 (5)	0	0	389 880 (1)
Agency for Healthcare Research and Quality	1 (1)	1 (8)	0	0	0	0	296 320 (<1)
Total	78 (100)	13 (17)	22 (28)	22 (28)	1 (1)	20 (26)	81 226 730 (100)

^a^ Grant types R03, R21, and R34.

^b^ Career Development Award.

^c^ Grant types F31, F32, K01, K23, and T32.

^d^ Grant type K24.

^e^ Grant types P20, R15, R24, R43, T37, U01, U13, U48, U54, and U58.

### Refugee Health Research by Location

**Figure 2.**
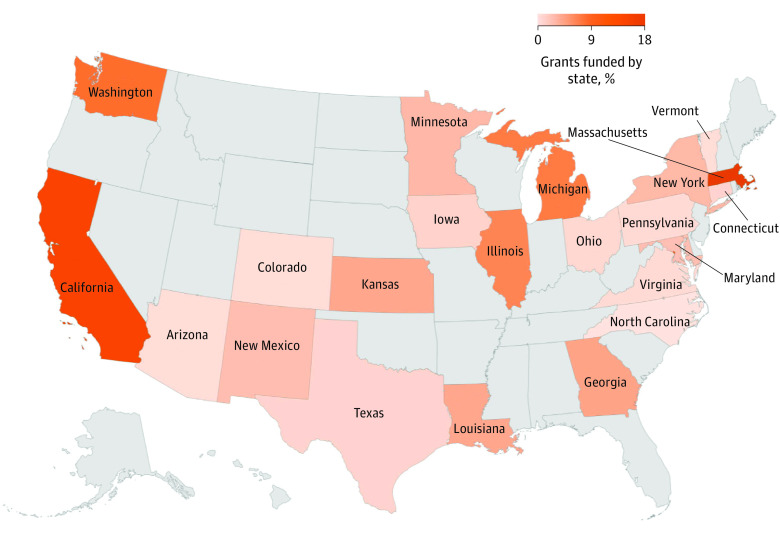
Percentage of Refugee Health Research Grants Funded by State

Most grants were US-based (60 grants [76%]). Overall, the state of Massachusetts received the highest amount of funds for refugee health research (18%), followed by California (15%) and Washington (9%) (Figure 2). Sixty grants (77%) funded refugee health research domestically and 18 grants (23%) were conducted outside of the US (eFigure 3 in Supplement 1).

### NIH Expenditure on Refugee Health Research

The total amount of funding for all 78 grants between 2000 and 2020 was $81.2 million (Table 2). Of this, $25.8 million (32%) was granted by NIMH and $18.4 million (23%) was granted by NIMHD (Table 2). This is in comparison with $5.4 trillion NIH funding spent from 2000 to 2020 on all research grants, research career awards, and training grants.^37^ Most recently in 2020, a total of about $2.3 million was invested by NIH in refugee health research, less than 0.01% of their $42 billion annual budget for medical research.^38^ Of the $2.3 million, 88% was funded by NICHD, and 71% was spent on refugee family dynamics and women’s and children’s health (including intergenerational studies). Overall, the median (IQR) amount funded through the 2-decade period was $491 314 ($291 708-1 530 130) (eTable 2 in Supplement 1). Individually, the year 2016 had the highest median (IQR) amount funded as compared with other years ($2 512 213 [990 189-3 383 886]) (eTable 2 in Supplement 1).

## Discussion

The forced displacement and refugee crisis is one of the defining public health challenges of our time. Spanning over 2 decades that witnessed multiple wars, natural disasters, a global pandemic, and economic inequality driving an unprecedented rate of mass displacement, our results highlight that this challenge is minimally addressed by scientific research funded through the NIH, a major public funder of biomedical research globally. Funding for refugee health research was consistently low and did not always align with the growing number of refugees resettled in the US. The cumulative number of refugees resettled over the years has grown, and given that the refugee lifetime experience is something that is carried with a person over the life course, NIH funding should have reflected that.

Our study also revealed that more than half of the refugee health research grants focused on mental health as their primary research without equal emphasis on other aspects of health among refugees, including chronic and infectious diseases.^39,40^ US-resettled refugees have a high burden of chronic noncommunicable disease and face major barriers to managing these conditions compared with native-born populations.^41,42,43,44^ Our study underscores the need to expand NIH-funded refugee health research beyond focusing only on mental health, which comprised the main topic area of almost half of all NIH-supported refugee health research. Although the mental health of refugees is important, the lack of funding for other aspects of well-being, particularly chronic illnesses, is concerning. The refugee population often has exposures that increase the risk of chronic illness and poor self-management behaviors or access to culturally concordant care, particularly as refugees age in exile.^45,46,47,48^ In fact, US health care spending on chronic manageable diseases such as hypertension and diabetes surpasses $600 billion annually and is a public health priority, including for refugee groups.^42,49,50^ Improving the management of chronic disease and barriers to health care among refugees could prevent inappropriate reliance on emergency departments or late presentation of disease that results in poor health outcomes and high costs to the health care system.^51^

We also found that most refugee health research grants were investigator-initiated research grants, followed by training grants, with few center grants, and 2 of the NIH institutes funded more than half of the grants. An increase in funding for refugee health research must involve all 28 centers and institutes to ensure a comprehensive understanding of refugees’ health needs and opportunities to develop interventions accordingly. Expanding the funding of refugee health with a focus on creating opportunities within each center or institute, including notices of special interests and opportunities that center community needs and priorities, would align with NIH’s recent intentions toward promoting health equity across its centers and institutes.^52,53^

Low levels of NIH investment in refugee health research may also be impacted by the lack of refugee representation among investigators or NIH reviewers or the lack of consideration of refugee investigators as minorities underrepresented in health sciences. Scientist-researcher pipeline programs may be useful in supporting investigators with lived refugee experience, who may have better access to target research populations to conduct this work in more professional capacities.^54,55^ Prior research has also identified a lack of diversity and expertise in health disparities research among reviewers in NIH peer review panels, which could contribute to the dearth of funding in this area.^56,57^ More work is also needed to understand how biases about refugees, influenced by negative media portrayal and political discourse around refugee populations,^13^ may translate into fewer allocated funds and stricter restrictions for refugee health research.

Our study also identified multiple grants that reflected a lack of knowledge or nuance regarding the terms used to describe forcibly displaced populations, confusing refugees with migrants and using these terms interchangeably, denoting a lack of understanding of the refugee and forced displacement experience. This is evident in the excluded abstracts which used words such as *refugees*, *immigrants*, or *migrants* interchangeably. Many grant abstracts in our sample used *refugee health* tags in their applications or keywords without including refugee populations in their study samples. Although other minoritized and migrant populations share similarities with refugee groups, and some research findings may be applicable across groups, there are specific aspects of forced displacement and its resettlement process that set this group apart, lead to particular care gaps, and dictate unique care needs.^58,59,60^ This highlights the need for NIH to invest in seminars, workshops, and language in their announcements that educate readers on the differences in terms and the importance of using the correct ones.

The NIH is well-positioned to lead the development of better interventions to improve the health of people who have refugee life experience and their families. A human rights perspective necessitates that we realize the inherent dignity of forcibly displaced people, including their right to health, and requires prompting research that addresses migration as a social determinant of health and leverages participatory action research methods to reduce existing disparities. Increased community-centered research is especially important as it integrates a human rights perspective that recognizes people as key actors in their own development, rather than passive recipients of service, thereby involving them in the research process as partners from inception to dissemination. Additional recommendations to expand the refugee health agenda include the following: first, solicitations of investigator-initiated research applications (R series grants) dedicated to refugee health (Table 3).^61^ Second, the NIH could develop incentive programs for early-stage researchers by establishing more training and career development awards specifically studying refugee health, especially in geographic areas with high rates of refugee resettlement such as California and Texas.^62^ Third, the NIH could support mentorship opportunities for refugee health mentors to train and support future researchers interested in refugee health research.^63,64^ Fourth, the NIH could partner with multilateral organizations such as the United Nations, International Monetary Fund, and the World Bank to fund refugee health research as these organizations are the majority contributors to international humanitarian efforts.^65^ Finally, the NIH could highlight current initiatives and new developments in refugee health research on its science highlights webpage to call attention to the important work being done in the field.^66^

**Table 3.** Recommendations for Immediate Action to Increase the National Institutes of Health (NIH) Support of Refugee Health Research

Recommendations	Targeted outcome
Dedicate funding that would directly impact refugee health and health care research across all 28 centers and institutes	This will help address the lower number of grants in research areas such as chronic disease. Involvement of all centers would ensure a more comprehensive understanding of refugee health and health care needs
Increase outreach efforts to attract more investigators and faculty that might be interested in refugee health research by including refugee health solicitations in R-series grant applications	Refugee health research solicitation through Notices of Special Interest and Requests for Applications that clearly define refugees and acknowledge them as a distinct research population underrepresented in health research
Incentivize upcoming researchers by establishing more training and career development awards specific to refugee health	Increasing mechanisms focusing on pipeline programs and capacity building to train investigators in refugee health would help attract more investigators and faculty to consider pursuing refugee health as their primary research area
Provide opportunities to graduate students or researchers with lived refugee experience interested in refugee health research	Clearly define researchers with a refugee experience as underrepresented in medical sciences who bring diverse perspectives to scientific research and provide eligibility to apply for diversity supplements
Partner with multilateral organizations	Develop international grant programs in collaboration with multilateral organizations such as the United Nations, International Monetary Fund, and the World Bank
Highlight the current initiatives and new developments in the study of refugee health	Conduct seminars and webinars disseminating results from NIH-funded researchers on refugee health and other communications from the NIH will help call attention to the crucial work being done by health care researchers to address policy issues and enhance care in refugee health

### Limitations

Despite our important findings, this study has some limitations. First, our search terms may have omitted grants related to refugee health research that were not captured by our search strategy or that were omitted from the NIH online reporting system. Moreover, the RePORT system only provides minimal information about the grant proposals, such as abstracts and keywords. Second, we do not know the total number of refugee health research applications submitted to the NIH that were not funded, given that the RePORT database only lists funded projects. It is also possible that high volumes of refugee health research applications were submitted, but few were actually funded. Third, this study does not capture changes beyond 2020 which future studies can include for a more comprehensive perspective. Potential changes following 2020 include mass displacement from the war in Ukraine, multiple wars in Israel-Palestine including the most recent October 2023 conflict in Gaza, and increased attention to health equity across NIH institutes followed by the murder of George Floyd, all of which could influence the refugee health research landscape. Although these limitations may have affected our final results, our methods and approach followed previously set guidelines on using RePORT data to assess NIH funding parity.^67^ Additionally, some contextual factors that could influence funding levels may exist (eg, NIH budget cuts) and can be examined in future research, but our study was limited to cross-sectional data to characterize overall NIH funding.

## Conclusions

To our knowledge, this is the first research paper determining the amount of NIH funding spent on refugee health research, a critical first step in assessing lacunae in refugee health research. The NIH must spearhead refugee health research in a new and accelerated direction in response to the unprecedented rates of forced displacement. Given the upcoming 2023 NIH budget is $62.5 billion (35% higher than the previous year), more money should be allocated to studying health disparities among refugees.^38^ Increased NIH funding for refugee health research would fill important research, clinical, and population health gaps as well as support the recruitment of new researchers invested in addressing health inequities in a rapidly growing US-based and global population.

## Notes

Supplement 1. **eTable 1. **Reasons for Exclusion of Grants With Keyword Matches With Examples **eTable 2. **Median and Interquartile Range (IQR) of Refugee Health Research Funding from 2000 to 2020 **eFigure 1. **STROBE Flow Chart **eFigure 2. **Average Duration of Grants in Years by Type of Grant **eFigure 3. **Percentage of Grants Funded by Location of Research

Supplement 2.
Data Sharing Statement
