# Supplementary material for: Funding for Refugee Health Research From the National Institutes of Health Between 2000 and 2020
Source: JAMA Netw Open. 2024 Jan 10;7(1):e2350837. doi: 10.1001/jamanetworkopen.2023.50837 (PMC10782235; doi:10.1001/jamanetworkopen.2023.50837)
Supplement: Supplement 1. — eTable 1. Reasons for Exclusion of Grants With Keyword Matches With Examples eTable 2. Median and Interquartile Range (IQR) of Refugee Health Research Funding from 2000 to 2020 eFigure 1. STROBE Flow Chart eFigure 2. Average Duration of Grants in Years by Type of Grant eFigure 3. Percentage of Grants Funded by Location of Research [file jamanetwopen-e2350837-s001.pdf]

Supplemental Online Content

Kaur M, Bridi L, Kaki D, et al. Funding for refugee health research from the National Institutes of Health between 2000-2020. *JAMA Netw Open*. 2024;7(1):e2350837. doi:10.1001/jamanetworkopen.2023.50837

eTable 1. Grants With Keyword Matches Excluded for not Directly Addressing the Health of Refugee Populations
eTable 2. Median and Interquartile Range (IQR) of Refugee Health Research Funding from 2000 to 2020
eFigure 1. STROBE Flow Chart
eFigure 2. Average Duration of Grants in Years by Type of Grant
eFigure 3. Percentage of Grants Funded by Location of Research

This supplemental material has been provided by the authors to give readers additional information about their work.

© 2024 Kaur M et al. *JAMA Network Open.*

eTable 1. Grants With Keyword Matches Excluded for not Directly Addressing the Health of Refugee Populations

**Reason for exclusion**	**Example grants (title)**
Study citing refugee health research conducted previously or using the keyword “refugee” but not actually studying refugees in the research aims of the current grant	• Diabetes and Heart Disease Risk in Blacks<br>• Improving Antibodies to Virion Based HIV Vaccines<br>• Training in Emerging Infectious Diseases<br>• Developing Community-Based Interventions for American Indian Mental Health<br>• Moralization, Risk Perceptions, and Smoking Cessation in the U.S. and Denmark<br>• Postdoctoral Training in Posttraumatic Stress Disorder<br>• Cholera in Goma, DRC
Interventions with potential refugee health applications but no refugee populations were planned to be included in the study aims	• Refugees and Immigrants 'New Ventures' E J Partnership<br>• Multilingual Health Education Resource Project<br>• EthnoMed Knowledge Management Grant<br>• Assessment of Dehydration in Children with Diarrhea in Resource-Limited Settings<br>• Genomics, Biometrics and Identity
Grants funding centers, cores, and conferences for refugee health but not directly doing refugee health research	• Children’s Hospital Center for Refugee Trauma<br>• Present and Future Research on Immigrant Children and Families<br>• Integrated Care for Asian Americans, Native Hawaiians, and Pacific Islanders: Making Research Work to Improve Our Health<br>• Outreach and Education Core<br>• Administrative Core
Keywords such as “asylum seekers”, “emigrant” and “‘migrants” present in the grant abstract but not related to these populations	• Guide to Substance Abuse and Mental Health Website<br>• Extra-Cytoplasmic Function Sigma Factor Senses and Responds to Beta-Lactam Stress in Gram-Positive Bacteria<br>• Ribonucleotide Reductase of the Malaria Parasite
Grants having no information available about the study aims on the NIH RePORT database	• Refugee Health Surveillance Program<br>• Advancing Treatment and Services for Refugee Children and Adolescents<br>• Trauma Informed Services for Refugee Children and Youth<br>• Promoting Refugee Wellbeing: A Community Intervention<br>• Descriptive Study of Unaccompanied Refugee Minors Program

© 2024 Kaur M et al. *JAMA Network Open.*

eTable 2. Median and Interquartile Range (IQR) of Refugee Health Research Funding from 2000 to 2020

Fiscal Year	Median	Interquartile Range (IQR)
2000-2020	$ 491,314.00	$ 1,238,422.00
2000	$ 764,248.50	$ 2,139,802.00
2001	$ 857,728.00	NA
2002	$ 247,682.50	$ 392,397.00
2003	$ 483,264.00	$ 713,566.00
2004	$ 475,012.00	$ 1,322,686.00
2005	$ 442,416.50	$ 1,716,623.50
2006	NA	NA
2007	$ 1,853,250.00	$ 556,500.00
2008	$ 624,249.00	$ 2,126,206.00
2009	$ 1,101,935.50	$ 1,330,637.50
2010	$ 1,722,395.50	$ 2,673,265.00
2011	$ 209,140.00	$ 174,360.00
2012	$ 1,046,700.00	$ 1,831,699.00
2013	$ 1,501,831.00	$ 748,116.00
2014	$ 412,347.50	$ 157,073.00
2015	$ 422,375.00	$ 891,431.50
2016	$ 2,512,213.00	$ 2,393,697.00
2017	$ 447,242.00	$ 674,355.00
2018	$ 394,453.00	$ 3,424,209.00
2019	$ 273,994.50	$ 274,001.50
2020	$ 389,880.00	$ 684,641.00

© 2024 Kaur M et al. *JAMA Network Open.*

eFigure 1. STROBE Flow Chart STROBE, Strengthening the Reporting of Observational Studies in Epidemiology.

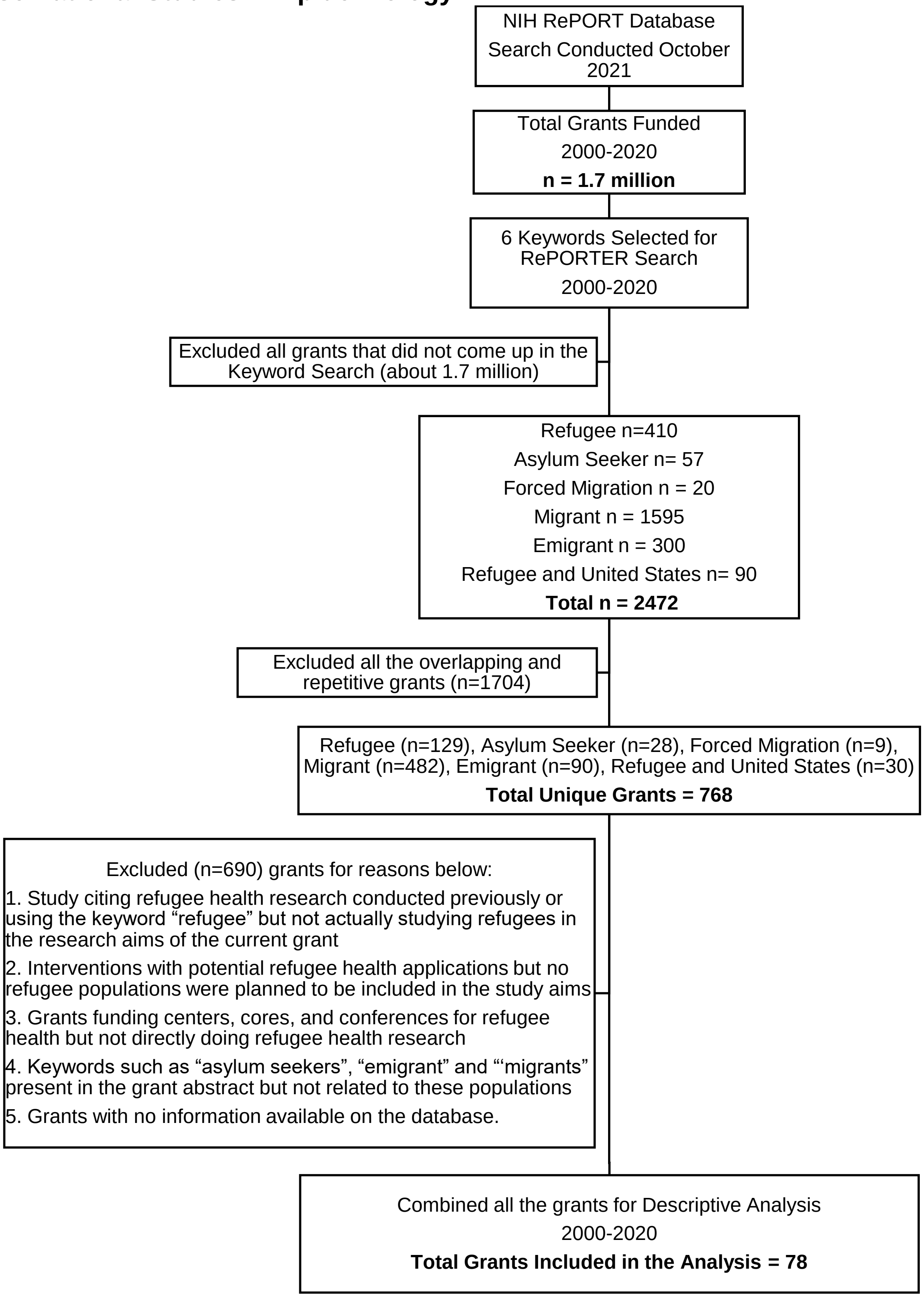

© 2024 Kaur M et al. *JAMA Network Open.*

eFigure 2. Average Duration of Grants in Years by Type of Grant

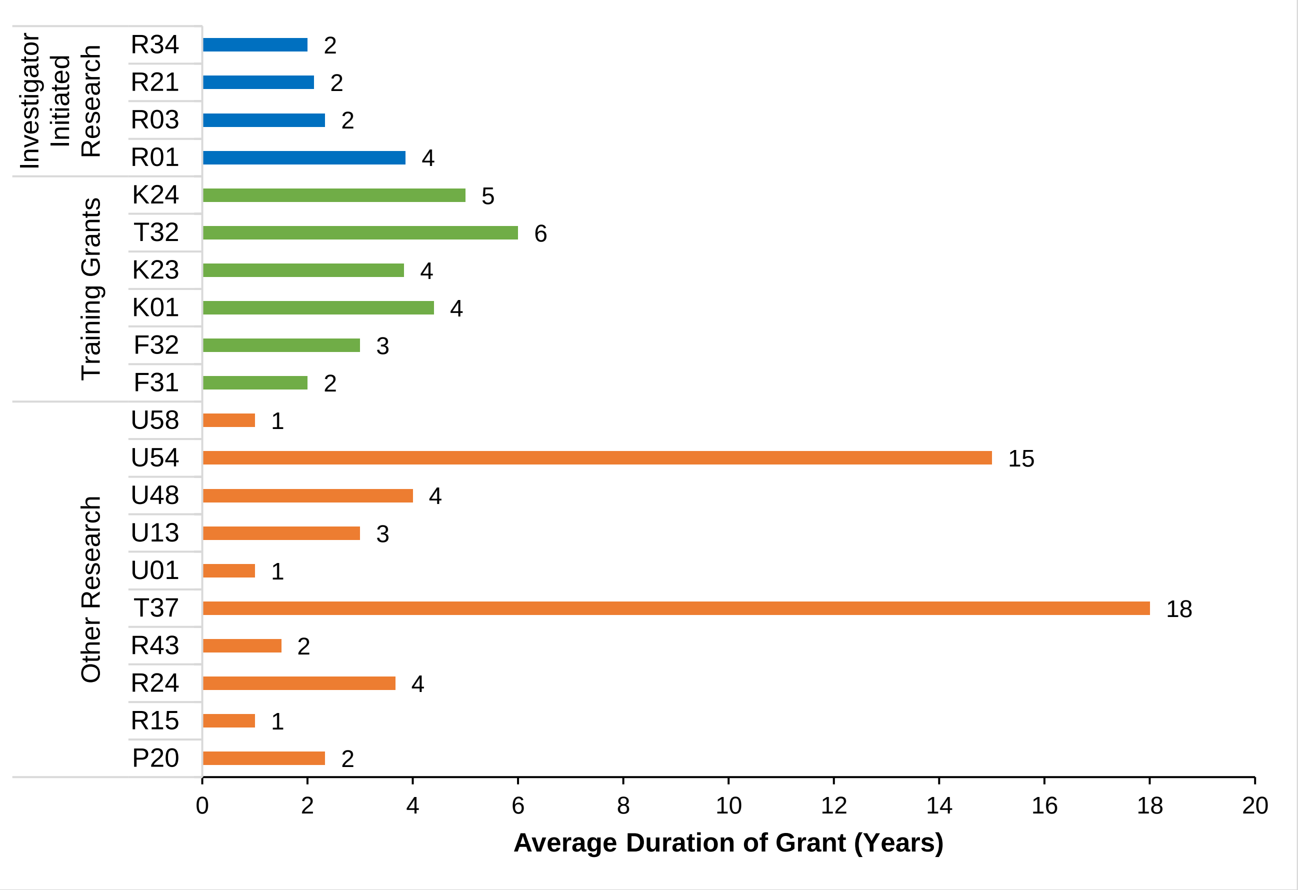

© 2024 Kaur M et al. *JAMA Network Open.*

eFigure 3. Percentage of Grants Funded by Location of Research

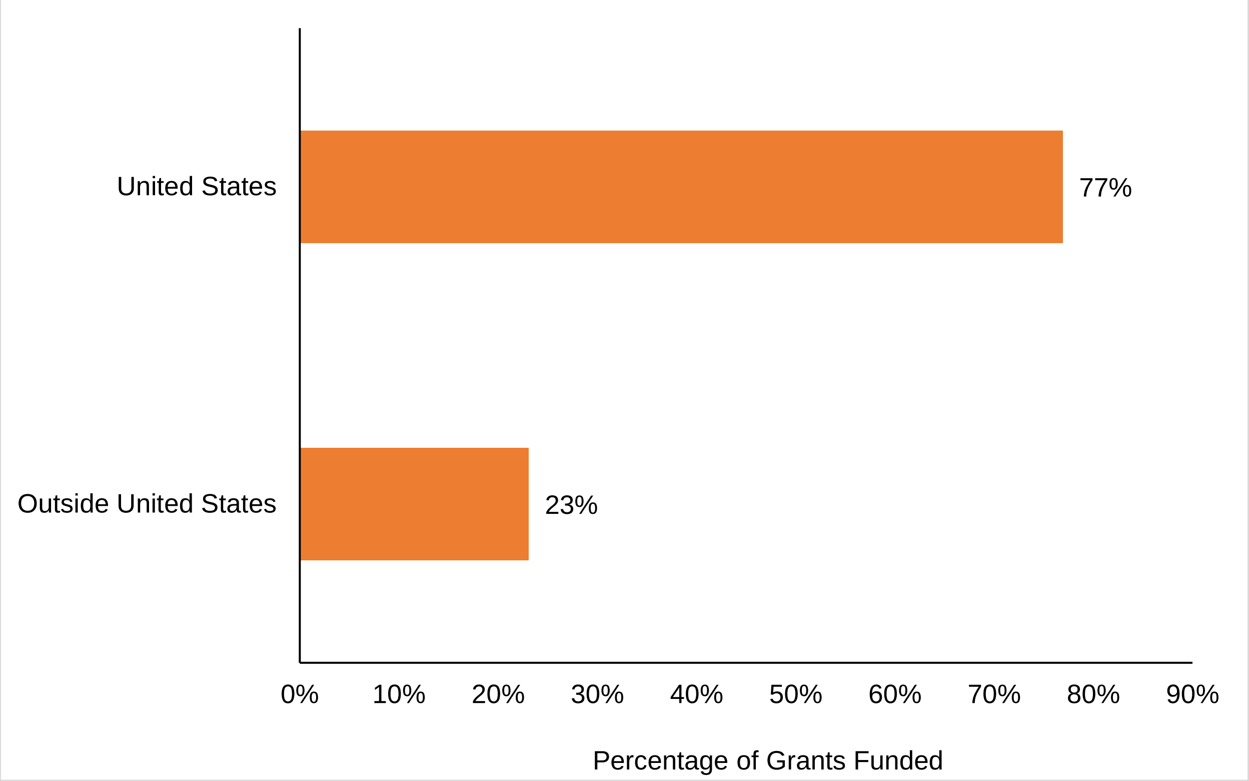

© 2024 Kaur M et al. *JAMA Network Open.*
