# Supplementary material for: Funding for Refugee Health Research From the National Institutes of Health Between 2000 and 2020
Source: JAMA Netw Open. 2024 Jan 10;7(1):e2350837. doi: 10.1001/jamanetworkopen.2023.50837 (PMC10782235; doi:10.1001/jamanetworkopen.2023.50837)
Supplement: Supplement 2. — Data Sharing Statement [file jamanetwopen-e2350837-s002.pdf]

Data Sharing Statement

Kaur. Funding for Refugee Health Research From the National Institutes of Health Between 2000 and 2020. *JAMA Netw Open*. Published January 09, 2024.
doi:10.1001/jamanetworkopen.2023.50837

Data
Data available: Yes
Data types: Data (not involving human participants)
How to access data: https://reporter.nih.gov/
When available: With publication

Supporting Documents
Document types: None

Additional Information
Who can access the data: anyone requesting the data
Types of analyses: for any purpose
Mechanisms of data availability: with investigator support,
